# Mediating effects of workgroup processes on the relationship between nurse turnover and nurse outcomes in hospitals

**DOI:** 10.3389/fpubh.2023.1255983

**Published:** 2023-11-23

**Authors:** Sung-Heui Bae, Suin Kim, Hwasook Myung

**Affiliations:** College of Nursing, Graduate Program in System Health Science and Engineering, Ewha Womans University, Seoul, Republic of Korea

**Keywords:** intent to leave, job satisfaction, nurse–nurse collaboration, nurse outcomes, nurse turnover, team cohesion, workgroup processes

## Abstract

**Background:**

Nurse turnover is often considered to be an outcome, and few studies have investigated its consequences in nursing care. The underlying mechanism of the nurse turnover–nurse outcome relationship has not been empirically investigated. Therefore, this study examines workgroup processes and nurse outcomes as the consequences of nurse turnover and the mediating effect of workgroup processes on the nurse turnover–nurse outcomes relationship.

**Methods:**

A cross-sectional design was adopted to investigate the data collected from 264 staff nurses. Furthermore, six-month turnover rates, workgroup processes (nurse–nurse collaboration, team cohesion), and nurse outcomes (job satisfaction, intent to leave) were utilized in the multivariate regression models.

**Results:**

Overall, 53 (24.4%) nurses had worked in nursing units with a zero six-month turnover rate. The average mean six-month turnover rate was 15.5%. Nurse turnover adversely affected nurses’ job satisfaction and several subscales of team cohesion including task cohesion and social cohesion. Team cohesion partially mediated the relationship between nurse turnover and job satisfaction.

**Conclusion:**

Nurse turnover decreased job satisfaction and team cohesion, and team cohesion partially mediated the nurse turnover–nurse outcomes relationship. These findings provide evidence supporting the significant adverse effects of nurse turnover and suggest the potential role of workgroup processes in explaining the underlying mechanism of the relationship between nurse turnover and nurse outcomes.

**Implications for nursing and health policy:**

Healthcare organizations must create a positive work environment to reduce nurse turnover. Further, states and countries should try to develop and establish nursing and health policies to prevent turnover.

## 1 Introduction

Many countries experience nursing shortages, which is a critical issue in nursing care (1, 2). The coronavirus disease 2019 (COVID-19) compounded the problem as it exacerbated work environments, affected nurses’ psychological and physical health, and increased their turnover intentions (3, 4), alongside creating a great need for nursing care. In this era, recruiting and retaining nurses has become even more important. Nurse turnover rates vary by country. In recent studies, annual nurse turnover rates were 27.1% in the US (5), 23% in Israel (6), and 19.7% in South Korea (7).

Nurse turnover is often considered to be an outcome. Studies have often focused on analyzing the factors that contribute to nurse turnover, and some have also examined its consequences (8). One previous study (9) found that nurse turnover adversely affects nurse outcomes, such as mental health and job satisfaction. Adverse patient outcomes, including pressure ulcers and medical errors (9, 10), also increase as nurse turnover increases. However, evidence regarding the impact of nurse turnover remains scarce. Moreover, the underlying mechanism of the relationship between nurse turnover and outcomes has not been examined (11).

Using McGrath’s (12) input-process-outcome (IPO) framework, Bae et al. (13) examined workgroup processes to explain how nurse turnover affects patient outcomes. They included workgroup cohesion, relationship coordination, and learning as workgroup processes. A decrease in workgroup learning was observed when nurse turnover increased. However, they found no evidence of workgroup processes being the underlying mechanism. Thus, further studying whether workgroup processes serve as the underlying mechanism of the impact of nurse turnover on outcomes is necessary.

Workgroup processes, which are defined as a mechanism that combines team members’ capabilities and behaviors, which have affective, behavioral, and cognitive domains (14). Collaboration and cooperation are considered to be behavioral workgroup processes (14). Collaboration has been described in healthcare as team members communicating well and supporting each other respectfully while working together (15–17). Further, better nurse–nurse collaboration is strongly related to higher job satisfaction (18, 19) and lower intention to leave (18). Team cohesion is classified as an affective workgroup process (14) and is defined as members’ commitment to a task (20). Among hospital nurses, team cohesion is negatively correlated with turnover intention (21). Moreover, team cohesion can improve nurse managers’ job satisfaction (22), which can decrease their intent to leave and affect the healthcare organization’s success (23, 24).

Nurse turnover may adversely affect both nurse–nurse collaboration and team cohesion. When a large number of nurses leave the unit, the remaining nurses doubt whether they should stay as well, which reduces their motivation and triggers additional turnover (25). Newcomer nurses may encounter ambiguous work contexts and require time to adapt to workgroup norms and communication patterns. Nurses working in units with frequent turnover need more time to adjust and supervise new nursing staff (13). When nurse turnover increases, nurse–nurse collaboration and team cohesion cannot easily be established in the unit. In such nursing units, the remaining nurses may be dissatisfied and become likely to leave. Through this underlying mechanism, workgroup processes, including nurse–nurse collaboration and team cohesion, may mediate the relationship between nurse turnover and nurse outcomes. However, the mediating effect of workgroup processes has not yet been comprehensively studied (11). Hence, it is worth examining the influence of nurse turnover on nurse outcomes as well as the mediating effect of workgroup processes on this relationship.

## 2 Materials and methods

### 2.1 Aim

**Figure 1:**
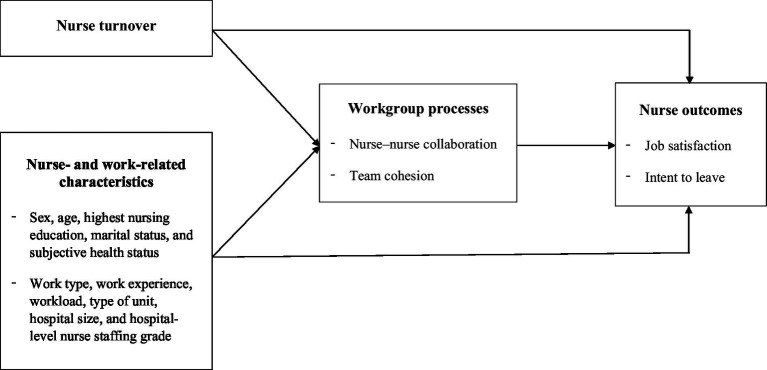
Conceptual model of the study.

Based on McGrath’s (12) IPO framework, this study aimed to examine the impact of nurse turnover on workgroup processes and nurse outcomes as well as the mediating effect of workgroup processes on that relationship. The workgroup processes, namely nurse–nurse collaboration and team cohesion, will provide not only the consequences of nurse turnover but also the mechanism underlying relationship between the nurse turnover and nurse outcomes (Figure 1).

### 2.2 Study design, sample, and data collection

A cross-sectional design was employed to analyze the impact of nurse turnover on workgroup processes and nurse outcomes as well as the mediating effects of workgroup processes on the relationship between nurse turnover and nurse outcomes. The convenience sampling method was used to collect the data from July 2022 to September 2022. The inclusion criteria were being nurses providing direct nursing care in medical or surgical units at acute care hospitals in South Korea and having worked for at least 6 months in the current units. Nurse managers at the same units were involved in a separate survey in order to acquire data of turnover rates.

The participants were asked to answer structured questionnaires, which were distributed separately to nurse managers and staff nurses via an online survey. For nurse managers, three additional questions were asked to measure the six-month turnover rates of nurses in their units. It took approximately 15 to 20 min for survey participants to complete the questionnaire and a mobile gift voucher was given to them. We contacted 270 hospitals with 201 to 1,000 beds nationwide, and 397 nurses at 35 hospitals responded. The inclusion criteria were as follows (a): working in medical/surgical units (b), working in the current unit for at least 6 months, and (c) who provided direct nursing care (bedside nurses only). The minimum sample size of 175 required for the multivariate regression analysis was calculated using an effect size of 0.15, 26 predictors, a significance level of 0.05, and a power of 0.80 in G^*^Power 3.1.9.4 (26). The sample size of this study was thus sufficient.

### 2.3 Measures

#### 2.3.1 Nurse-and work-related characteristics

Sex, age, highest nursing education, marital status, and self-reported health status were analyzed as the demographic characteristics of nurses working in medical or surgical units. Work-related characteristics included position type, work type, work experience in current hospitals, workload, type of unit, hospital size, and hospital-level nurse staffing grade. The amount of performance that is required for a job is workload (27, 28). Workload was measured with four items assessing performance and frequency of work from 1 (“never”) to 6 (“5 or more days a week”); the total scores ranged from 4 to 24 points. The Cronbach’s alpha in this study was 0.83, and higher scores represent a greater workload. The hospital-level nurse staffing grade was based on the nurse-to-patient ratio at the hospital level; first grade represented better nurse staffing.

#### 2.3.2 Nurse turnover

The six-month turnover rates of nurses in a nursing unit were measured in response to three questions asking about the number of nurses working at two certain points in time and the number of nurses leaving the units during that period. Nurse managers answered how many nurses were working from January 1, 2022 (time A) to June 30, 2022 (time B) and how many nurses left their units between times A and B. This period was approximately 6 months before data collection. Based on this information, the six-month turnover rates were calculated by dividing the number of nurses who resigned between times A and B by the average number of nurses working at times A and B. Nurse managers responded to the turnover rate. This turnover rate was applied to the nurses in the same unit. Based on the distribution of turnover, the six-month turnover rates were grouped into zero (0%), low (1–14%), moderate (15–23%), and high (24–50%) turnover levels. The estimation of nurse turnover rate based on nurse managers’ reports has been used in a previous study (13).

#### 2.3.3 Workgroup processes

Collaboration between nurses was measured using the nurse–nurse collaboration scale developed by Dougherty and Larson (29) and modified by Lee and Hwang (30). The instrument contains five subscales and 35 items: “conflict management” (seven items), “communication” (eight items), “shared process” (eight items), “coordination” (five items), and “professionalism” (seven items). A four-point Likert-type scale was used, with scores ranging from 1 (“strongly disagree”) to 4 (“strongly agree”). Moreover, seven items (5–7, 12–15) were reverse coded, and higher scores indicate that nurse–nurse collaboration between nurses is more positive. The Cronbach’s alphas were 0.89 when it was developed (29) and 0.87 in a previous study (30). In this study, the Cronbach’s alpha was 0.91. Table 1 presents the Cronbach’s alpha for each subscale.

**Table 1:** General characteristics of the study variables (*N* = 264).

Variables	*n* (%)	Mean (SD)	Cronbach’s alpha
Nurse turnover			
Six-month turnover rate	*N* = 217	15.49 (14.20)	
0%	53 (24.4)		
1–14%	50 (23.1)		
15–23%	71 (32.7)		
24–50%	43 (19.8)		
Work group processes			
Nurse–nurse collaboration	*N* = 227	2.83 (0.30)	0.91
Conflict management	*N* = 238	2.88 (0.45)	0.84
Communication	*N* = 239	2.56 (0.34)	0.63
Shared process	*N* = 234	2.86 (0.36)	0.77
Coordination	*N* = 240	2.85 (0.42)	0.66
Professionalism	*N* = 239	3.05 (0.46)	0.92
Team cohesion	*N* = 244	5.55 (1.16)	0.77
Task cohesion	*N* = 247	6.04 (1.40)	0.70
Social cohesion	*N* = 246	5.16 (1.58)	0.75
Individual attraction to the group	*N* = 247	5.35 (1.71)	0.24
Nurse outcomes			
Job satisfaction	*N* = 235	2.46 (0.56)	0.86
Intent to leave	*N* = 237	3.75 (0.87)	0.86
Nurse- and work-related characteristics			
Sex	*N* = 234		
Male	5 (2.1)		
Female	229 (97.9)		
Age, years in 2022	*N* = 231	32.58 (7.97)	
21–30	121 (52.4)		
31–40	67 (29.0)		
41–50	32 (13.9)		
> = 51	11 (4.7)		
Highest nursing education	*N* = 230		
Associate’s degree	41 (17.8)		
Bachelor’s degree	178 (77.4)		
Master’s degree or PhD in nursing	11 (4.8)		
Marital status	*N* = 233		
Married	90 (38.6)		
Widowed, divorced, separated	5 (2.2)		
Never married	138 (59.2)		
Subjective health status	*N* = 231		
Very good	20 (8.7)		
Good	52 (24.7)		
Fair	111 (48.0)		
Poor	43 (18.6)		
Work type	*N* = 264		
Three-shifts rotation	239 (90.5)		
Other	25 (9.5)		
Work experience in current hospital (years)	*N* = 234	7.62 (7.18)	
Under 1 year	16 (6.8)		
1 year to under 3 years	54 (23.1)		
3 years to under 5 years	37 (15.8)		
5 years to under 10 years	60 (25.6)		
10 years or over	67 (28.6)		
Workload	*N* = 263	18.05 (3.60)	0.83
Type of unit	*N* = 264		
Medical	77 (29.2)		
Surgical	97 (36.7)		
Medical-surgical	90 (34.1)		
Hospital size (beds)	*N* = 264	372.65 (115.03)	
201–300	1,063 (40.2)		
301–400	49 (18.6)		
401–500	83 (31.4)		
501–1,000	26 (9.8)		
Hospital-level nurse staffing grade	*N* = 264		
1^st^ grade	251 (95.1)		
2^nd^ to 5^th^ grade	13 (4.9)		

SD (standard deviation); PhD (Doctor of Philosophy).

Team cohesion in the nursing units was measured using the team cohesion scale developed by Carless and De Paola (31) and modified by Kang and You (32). The instrument contains three subscales and 10 items: “task cohesion” (four items), “social cohesion” (four items), and “individual attraction to the group” (two items). A nine-point Likert-type scale was used, with scores ranging from 1 (“strongly disagree”) to 9 (“strongly agree”); six items (2–4, 6–8) were reverse coded. The total mean score ranged from 1 to 9 points. The higher scores indicate greater team cohesion. The reliability of the instrument measured with Cronbach’s alpha was 0.78 in a previous study (32) and 0.77 in this study.

#### 2.3.4 Nurse outcomes

Job satisfaction was measured using items from the Korean version of the Copenhagen Psychosocial Questionnaire Scale (COPSOQ-K), an instrument modified from COPSOQ II by June and Choi (33). The scale comprises four items (e.g., “To what extent are you satisfied with your career prospects?”). A four-point Likert-type scale was used for this scale, with scores ranging from 1 (“very unsatisfied”) to 4 (“very satisfied”). The total score ranged from 1 to 4 points. Higher scores mean greater job satisfaction. The Cronbach’s alphas at the time of development (33) and in this study were 0.78 and 0.86, respectively.

Nurses’ intent to leave was measured by Lawler’s (34) tool for turnover intention, which was modified for nurses by Park et al. (35). This scale comprises four items (e.g., “I sometimes think of leaving my current workplace”). Nurses were asked to indicate how much they agree with their intention of turnover in each statement. A five-point Likert-type scale was used, with scores ranging from 1 (“strongly disagree”) to 5 (“strongly agree”). The total score ranged from 1 to 5 points. Higher scores indicate greater turnover intention. The Cronbach’s alpha was 0.91 in a previous study (35) and 0.86 in this study.

### 2.4 Data analysis

All the data were analyzed using SAS version 9.4. Descriptive statistics, including mean, standard deviation, frequency, and percentage, were evaluated for each variable. Multivariate regression was used to estimate the effects of nurse turnover on workgroup processes and nurse outcomes. The mediating effect of workgroup processes on the relationship between nurse turnover and nurse outcomes was also analyzed using multivariate regression. A significance level of 0.05 was applied. To construct the analytic model, nurse- and work-related characteristics were included as control variables. Dummy variables of categorical variables, such as sex, highest nursing education, and subjective health status, were utilized. Continuous variables such as age and workload were also included in the analytic model.

### 2.5 Ethical considerations

This study was conducted with the approval of the university’s Institutional Review Board (approval no. XXX-202205-0005-01), and informed consent was obtained from all participants via online. Permission to use the instruments was provided from the authors, allowing us to use the instruments employed in this study.

## 3 Results

### 3.1 Participant characteristics

Among the 397 participants, 29 who did not work in their current units for at least 6 months were excluded from the study, and 45 participants were excluded because they did not work in either the medical or surgical units. Among the remaining 323 participants, 26 participants did not answer more than two-thirds of the questions, including the nurse turnover, workgroup processes, and nurse outcome questions; therefore, they were excluded. Another 33 nurse managers were also excluded because they did not provide direct nursing care. Finally, 264 responses from staff nurses working in 28 hospitals were utilized for the analysis. Non-missing totals were used in the analysis without imputation; hence, the total number of questions varied from question to question.

Table 1 presents the characteristics of the 264 nurses. In total, 53 (24.4%) nurses worked in nursing units with a zero six-month turnover rate and the average six-month turnover rate was 15.5%. Mean nurse–nurse collaboration was 2.83 ± 0.30, and mean team cohesion was 5.55 ± 1.16. The participants’ mean job satisfaction was 2.46 ± 0.56, and mean intent to leave was 3.75 ± 0.87. The nurses’ mean age was 32.58 ± 7.97 years and 38.6% of them were married. Additionally, more than half (54.2%) had 5 years or more work experience in their current hospitals and mean perceived workload was 18.05 ± 3.60.

### 3.2 Effects of nurse turnover and workgroup processes on nurse outcomes

Multivariate regression was used to examine the effects of nurse turnover and workgroup processes on nurses’ outcomes (Tables 2, 3). Nurse outcomes included job satisfaction (Models 1–7) and intention to leave (Models 8–14). Compared with the zero six-month turnover rate, higher nurse turnover (24–50%) in nursing units decreased nurses’ job satisfaction (*β* = −0.366, standard error [SE] = 0.136) with an adjusted R-squared of 0.160. Nurse–nurse collaboration also increased job satisfaction (*β* = 0.944, SE = 0.116) with an adjusted R-squared of 0.358. When both nurse turnover and nurse–nurse collaboration were added into the model, the effect of nurse turnover on job satisfaction decreased (*β* = −0.304, SE = 0.122) whereas the effect of nurse–nurse collaboration on job satisfaction slightly increased (*β* = 0.946, SE = 0.125) with an adjusted R-squared of 0.367. Among these subscales, communication (*β* = 0.322, SE = 0.124) and professionalism (*β* = 0.300, SE = 0.113) were positively associated with job satisfaction with an adjusted R-squared of 0.364. Team cohesion also increased job satisfaction (*β* = 0.130, SE = 0.031) with an adjusted R-squared of 0.215. When nurse turnover and team cohesion were included in the model, the effects of nurse turnover (*β* = −0.293, SE = 0.133) and team cohesion (*β* = 0.126, SE = 0.033) on job satisfaction decreased with an adjusted R-squared of 0.222. Among the three subscales of team cohesion, only task cohesion was positively associated with job satisfaction (*β* = 0.067, SE = 0.031) with an adjusted R-squared of 0.215.

**Table 2:** Nurse turnover and workgroup processes contributing to job satisfaction.

Variables	Model 1	Model 2	Model 3	Model 4	Model 5	Model 6	Model 7
	β (SE)	β (SE)	β (SE)	β (SE)	β (SE)	β (SE)	β (SE)
Intercept	2.908 (0.445)^**^	−0.053 (0.494)	−0.019 (0.553)	−0.011 (0.596)	1.956 (0.435)^**^	1.985 (0.494)^**^	1.935 (0.503)^**^
Nurse turnover							
Six-month turnover rate (ref: 0%)							
1–14%	−0.074 (0.123)		−0.093 (0.110)	−0.069 (0.112)		−0.015 (0.121)	−0.030 (0.124)
15–23%	−0.080 (0.123)		−0.050 (0.118)	−0.030 (0.119)		−0.025 (0.130)	−0.016 (0.133)
24–50%	−0.366 (0.136)^**^		−0.304 (0.122)^**^	−0.313 (0.126)^*^		−0.293 (0.133)^*^	−0.288 (0.134)^*^
Workgroup processes							
Nurse–nurse collaboration		0.944 (0.116)^**^	0.946 (0.125)^**^				
Conflict management				0.031 (0.100)			
Communication				0.322 (0.124)^*^			
Shared process				0.143 (0.134)			
Coordination				0.160 (0.106)			
Professionalism				0.300 (0.113)^**^			
Team cohesion					0.130 (0.031)^**^	0.126 (0.033)^**^	
Task cohesion							0.067 (0.031)^*^
Social cohesion							0.034 (0.030)
Individual attraction to the group							0.029 (0.025)
Nurse- and work-related characteristics							
Sex (ref: Female)							
Male	0.097 (0.247)	0.062 (0.214)	0.051 (0.215)	−0.016 (0.224)	0.150 (0.237)	0.131 (0.239)	0.109 (0.244)
Age, years in 2022	<−0.001 (0.010)	<0.001 (0.008)	0.005 (0.009)	0.004 (0.009)	<−0.001 (0.009)	0.003 (0.010)	0.004 (0.010)
Highest nursing education (ref: Associate’s degree)							
Bachelor’s degree	−0.106 (0.104)	−0.036 (0.084)	−0.025 (0.093)	−0.021 (0.095)	−0.044 (0.091)	−0.050 (0.102)	−0.043 (0.103)
Master’s degree or PhD in nursing	0.107 (0.221)	0.130 (0.184)	0.012 (0.203)	0.125 (0.204)	0.022 (0.203)	0.164 (0.214)	0.170 (0.217)
Marital status (ref: Married)							
Widowed, divorced, separated	−0.257 (0.418)	−0.383 (0.282)	−0.266 (0.364)	−0.226 (0.367)	−0.499 (0.315)	−0.320 (0.404)	−0.325 (0.406)
Never married	0.225 (0.103)^*^	0.099 (0.085)	0.109 (0.092)	0.081 (0.094)	0.212 (0.092)^*^	0.206 (0.100)^*^	0.204 (0.102)^*^
Subjective health status (ref: Poor)							
Very good	0.438 (0.166)^**^	0.112 (0.141)	0.102 (0.152)	0.085 (0.153)	0.319 (0.151)^*^	0.369 (0.161)^*^	0.373 (0.163)^*^
Good	0.428 (0.121)^**^	0.287 (0.103)^**^	0.302 (0.110)^**^	0.294 (0.110)^**^	0.318 (0.111)^**^	0.353 (0.118)^**^	0.350 (0.119)^**^
Fair	0.288 (0.106)^**^	0.181 (0.088)^*^	0.185 (0.097)	0.161 (0.097)	0.238 (0.095)^*^	0.264 (0.103)^*^	0.258 (0.105)^*^
Work type (ref: Other)							
Three-shift rotation	0.031 (0.151)	0.080 (0.123)	0.063 (0.135)	0.067 (0.138)	0.011 (0.136)	0.010 (0.146)	0.009 (0.147)
Work experience in current hospitals	0.008 (0.010)	0.002 (0.008)	<−0.001 (0.009)	<−0.001 (0.009)	0.010 (0.009)	0.005 (0.010)	0.004 (0.011)
Workload	−0.036 (0.011)^**^	−0.025 (0.010)^**^	−0.026 (0.010)^*^	−0.025 (0.010)^*^	−0.031 (0.010)^**^	−0.032 (0.011)^**^	−0.032 (0.011)^**^
Type of unit (ref: Medical-surgical)							
Medical	0.043 (0.106)	−0.018 (0.084)	−0.034 (0.095)	−0.025 (0.096)	0.025 (0.091)	−0.010 (0.103)	−0.008 (0.104)
Surgical	0.013 (0.105)	0.072 (0.080)	0.062 (0.094)	0.071 (0.095)	0.031 (0.087)	0.010 (0.102)	0.022 (0.104)
Hospital size (ref: 201–300)							
301–400	0.081 (0.140)	0.066 (0.099)	0.028 (0.1239)	−0.012 (0.127)	0.085 (0.107)	0.034(0.135)	0.045 (0.138)
401–500	−0.274 (0.119)^*^	−0.152 (0.083)	−0.209 (0.107)	−0.241 (0.109)^*^	−0.179 (0.092)	−0.241 (0.116)^*^	−0.236 (0.118)^*^
501–1,000	0.046 (0.151)	−0.015 (0.120)	0.029 (0.135)	−0.008 (0.139)	0.051 (0.131)	0.094 (0.147)	0.098 (0.148)
Hospital-level nurse staffing grade (ref: 1^st^ grade)							
2^nd^ to 5^th^ grades	−0.314 (0.192)	−0.015 (0.154)	−0.097 (0.173)	−0.080 (0.174)	−0.213 (0.167)	−0.269 (0.186)	−0.263 (0.187)
Adj. R^2^	0.160	0.358	0.367	0.364	0.215	0.222	0.215
F test	2.73	7.13	5.76	4.98	4.12	3.45	3.15
*p*-value	0.0002	<0.0001	<0.0001	<0.0001	< 0.0001	< 0.0001	< 0.0001
*N*	192	210	182	182	218	190	190

SE (standard errors); PhD (Doctor of Philosophy).

* *p* < 0.05.

** *p* < 0.01.

**Table 3:** Nurse turnover and workgroup processes contributing to intent to leave.

Variables	Model 8	Model 9	Model 10	Model 11	Model 12	Model 13	Model 14
	β (SE)	β (SE)	β (SE)	β (SE)	β (SE)	β (SE)	β (SE)
Intercept	3.322 (0.702)^**^	6.010 (0.847)^**^	6.091 (0.969)^**^	5.860 (1.046)^**^	4.318 (0.682)^**^	4.439 (0.790)^**^	4.363 (0.799)^**^
Nurse turnover							
Six-month turnover rate (ref: 0%)							
1–14%	0.033 (0.195)		0.005 (0.193)	0.024 (0.196)		−0.038 (0.194)	−0.060 (0.198)
15–23%	0.077 (0.209)		0.057 (0.205)	0.047 (0.208)		0.010 (0.206)	0.068 (0.209)
24–50%	0.398 (0.213)		0.329 (0.212)	0.377 (0.219)		0.295 (0.211)	0.312 (0.211)
Work group processes							
Nurse–nurse collaboration		−0.941 (0.200)^**^	−0.911 (0.219)^**^				
Conflict management				−0.241 (0.175)			
Communication				−0.286 (0.218)			
Shared process				−0.178 (0.235)			
Coordination				0.121 (0.187)			
Professionalism				−0.286 (0.199)			
Team cohesion					−0.155 (0.049)^**^	−0.151 (0.052)^**^	
Task cohesion							−0.020 (0.050)
Social cohesion							−0.058 (0.048)
Individual attraction to the group							−0.076 (0.040)
Nurse- and work-related characteristics							
Sex (ref: Female)							
Male	0.109 (0.391)	0.132 (0.367)	0.175 (0.378)	0.101 (0.393)	0.017 (0.373)	0.072 (0.384)	0.106 (0.390)
Age, years in 2022	−0.011 (0.016)	−0.006 (0.014)	−0.014 (0.016)	−0.014 (0.016)	−0.009 (0.014)	−0.016 (0.016)	−0.016 (0.016)
Highest nursing education (ref: Associate’s degree)							
Bachelor’s degree	0.308 (0.165)	0.205 (0.144)	0.269 (0.164)	0.288 (0.167)	0.165 (0.144)	0.230 (0.164)	0.260 (0.166)
Master’s degree or PhD in nursing	−0.070 (0.350)	−0.227 (0.316)	−0.117 (0.357)	−0.084 (0.359)	−0.253 (0.320)	−0.131 (0.344)	−0.079 (0.346)
Marital status (ref: Married)							
Widowed, divorced, separated	−0.545 (0.661)	−0.197 (0.485)	−0.548 (0.638)	−0.501 (0.645)	−0.028 (0.497)	−0.460 (0.649)	−0.446 (0.649)
Never married	−0.362 (0.162)^*^	−0.294 (0.145)^*^	−0.276 (0.161)	−0.265 (0.164)	−0.379 (0.144)^**^	−0.339 (0.159)^*^	−0.306 (0.162)
Subjective health status (ref: Poor)							
Very good	−0.627 (0.263)^*^	−0.247 (0.243)	−0.250 (0.266)	−0.211 (0.270)	−0.495 (0.239)^*^	−0.542 (0.259)^*^	−0.500 (0.261)
Good	−0.645 (0.191)^**^	−0.435 (0.176)^*^	−0.507 (0.192)^**^	−0.520 (0.193)^**^	−0.477 (0.174)^**^	−0.556 (0.189)^**^	−0.540 (0.190)^**^
Fair	−0.493 (0.169)^**^	−0.375 (0.153)^*^	−0.348 (0.170)^*^	−0.354 (0.171)^*^	−0.482 (0.150)^**^	−0.470 (0.166)^**^	−0.452 (0.168)^**^
Work type (ref: Other)							
Three-shift rotation	−0.004 (0.240)	0.047 (0.210)	−0.094 (0.237)	−0.050 (0.243)	0.138 (0.213)	0.011 (0.235)	−0.007 (0.235)
Work experience in current hospitals	0.007 (0.016)	0.002 (0.014)	0.016 (0.016)	0.017 (0.016)	−0.004 (0.014)	0.010 (0.017)	−0.014 (0.017)
Workload	0.052 (0.018)^**^	0.044 (0.016)^**^	0.040 (0.018)^*^	0.042 (0.018)^*^	0.051 (0.016)^**^	0.047 (0.018)^**^	0.048 (0.018)^**^
Type of unit (ref: Medical-surgical)							
Medical	0.079 (0.165)	0.135 (0.144)	0.222 (0.165)	0.230 (0.167)	0.063 (0.143)	0.145 (0.164)	0.138 (0.164)
Surgical	0.149 (0.165)	0.050 (0.137)	0.111 (0.164)	0.135 (0.166)	0.082 (0.136)	0.143 (0.163)	0.163 (0.165)
Hospital size (ref: 201–300)							
301–400	−0.039 (0.221)	−0.036 (0.169)	−0.069 (0.216)	−0.105 (0.224)	−0.076 (0.169)	−0.084 (0.218)	−0.102 (0.221)
401–500	0.151 (0.188)	0.096 (0.142)	0.090 (0.187)	0.088 (0.192)	0.098 (0.144)	0.103 (0.186)	0.069 (0.189)
501–1,000	−0.289 (0.239)	−0.234 (0.207)	−0.301 (0.238)	−0.302 (0.245)	−0.317 (0.207)	−0.368 (0.236)	−0.396 (0.237)
Hospital-level nurse staffing grade (ref: 1^st^ grade)							
2^nd^ to 5^th^ grades	0.530 (0.303)	0.261 (0.264)	0.330 (0.302)	0.327 (0.305)	0.438 (0.264)	0.478 (0.298)	0.486 (0.298)
Adj. R^2^	0.095	0.190	0.168	0.160	0.151	0.131	0.130
F test	1.97	3.60	2.67	2.34	3.04	2.31	2.19
*p*-value	0.0097	<0.0001	0.0002	0.0007	<0.0001	0.0015	0.0021
N	194	212	184	184	220	192	192

SE (standard errors); PhD (Doctor of Philosophy).

* *p* < 0.05.

** *p* < 0.01.

Models 8 to 14 present the effects of nurse turnover and workgroup processes on intent to leave. Nurse turnover in nursing units did not affect intent to leave. The total score for nurse–nurse collaboration decreased nurses’ intent to leave (*β* = −0.941, SE = 0.200) with an adjusted R-squared of 0.190, and team cohesion also decreased intent to leave (*β* = −0.155, SE = 0.049) with an adjusted R-squared of 0.160. However, none of the subscales were related to intent to leave individually.

Both job satisfaction and intent to leave were significantly affected by nurses’ subjective health status. Nurses with better health conditions reported higher job satisfaction and lower intent to leave than those with poor health condition. Perceived workload impacted both job satisfaction and intent to leave; thus, a higher workload decreased job satisfaction and increased intent to leave.

### 3.3 Effects of nurse turnover on workgroup processes

Table 4 shows the effects of nurse turnover on nurse–nurse collaboration. Both the total (Model 15) and the subscale models (Models 16–20) were examined. Nurse turnover was only significantly related to the coordination subscale. Compared with the zero six-month turnover rate, higher nurse turnover decreased coordination (*β* = −0.210, SE = 0.106) with an adjusted R-squared of 0.047. However, it was not related to nurse–nurse collaboration or any of the other subscales. Subjective health status was positively related to nurse–nurse collaboration and several subscales (conflict management, communication, and professionalism). Longer work experience in the current hospitals also increased nurse–nurse collaboration. Furthermore, a greater workload adversely affected communication.

**Table 4:** Nurse turnover contributing to nurse–nurse collaboration.

Variables	Total	Conflict management	Communication	Shared process	Coordination	Professionalism
	Model 15	Model 16	Model 17	Model 18	Model 19	Model 20
	β (SE)	β (SE)	β (SE)	β (SE)	β (SE)	β (SE)
Intercept	3.141 (0.245)^**^	3.128 (0.382)^**^	2.867 (0.280)^**^	3.311 (0.294)^**^	3.472 (0.349)^**^	3.107 (0.382)^**^
Nurse turnover						
Six-month turnover rate (ref: 0%)						
1–14%	−0.037 (0.069)	0.073 (0.106)	−0.027 (0.078)	−0.041 (0.083)	−0.092 (0.097)	−0.045 (0.106)
15–23%	−0.060 (0.074)	−0.024 (0.113)	−0.084 (0.083)	0.002 (0.089)	−0.044 (0.104)	−0.070 (0.114)
24–50%	−0.063 (0.076)	−0.082 (0.116)	−0.089 (0.085)	−0.005 (0.091)	−0.210 (0.106)^*^	0.086 (0.116)
Nurse- and work-related characteristics						
Sex (ref: Female)						
Male	0.043 (0.136)	−0.358 (0.213)	−0.132 (0.156)	0.233 (0.163)	0.250 (0.195)	0.298 (0.213)
Age, years in 2022	−0.007 (0.006)	−0.009 (0.009)	−0.006 (0.006)	−0.010 (0.007)	−0.006 (0.008)	−0.006 (0.009)
Highest nursing education (ref: Associate’s degree)						
Bachelor’s degree	−0.067 (0.059)	−0.100 (0.091)	−0.125 (0.066)	−0.022 (0.070)	−0.156 (0.082)	−0.049 (0.090)
Master’s degree or PhD/DNP in nursing	−0.057 (0.128)	0.024 (0.191)	0.015 (0.139)	−0.127 (0.154)	−0.122 (0.174)	−0.053 (0.190)
Marital status (ref: Married)						
Widowed, divorced, separated	−0.001 (0.229)	0.287 (0.359)	0.125 (0.263)	−0.206 (0.276)	−0.104 (0.329)	−0.150 (0.359)
Never married	0.120 (0.057)	0.081 (0.089)	0.139 (0.065)^*^	0.073 (0.068)	0.108 (0.081)	0.232 (0.088)^**^
Subjective health status (ref: Poor)						
Very good	0.563 (0.092)^**^	0.426 (0.143)^**^	0.348 (0.104)^**^	0.188 (0.110)	0.235 (0.131)	0.541 (0.143)^**^
Good	0.132 (0.068)^*^	0.080 (0.104)	0.110 (0.076)	0.145 (0.081)	0.151 (0.095)	0.185 (0.105)
Fair	0.096 (0.060)	0.110 (0.092)	0.107 (0.067)	0.053 (0.072)	0.106 (0.084)	0.145 (0.092)
Work type (ref: Other)						
Three-shift rotation	−0.069 (0.085)	0.011 (0.130)	0.085 (0.096)	−0.210 (0.102)	−0.162 (0.119)	−0.169 (0.130)
Work experience in current hospitals	0.011 (0.006)	0.015 (0.009)	0.009 (0.007)	0.011 (0.007)	0.008 (0.008)	0.014 (0.009)
Workload	−0.011 (0.006)	−0.006 (0.010)	−0.016 (0.007)^*^	−0.006 (0.008)	−0.017 (0.009)	−0.005 (0.010)
Type of unit (ref: Medical-surgical)						
Medical	0.0946(0.059)	0.212 (0.091)^*^	0.131 (0.066)^*^	−0.008 (0.070)	0.060 (0.082)	0.063 (0.090)
Surgical	−0.053 (0.059)	0.013 (0.091)	−0.052 (0.066)	−0.073(0.070)	−0.127 (0.082)	−0.059 (0.090)
Hospital size (ref: 201–300)						
301–400	0.087 (0.077)	−0.124 (0.121)	−0.007 (0.088)	0.160 (0.093)	0.179 (0.110)	0.220 (0.120)
401–500	−0.044 (0.067)	−0.187 (0.102)	−0.076 (0.075)	−0.041 (0.080)	−0.030 (0.093)	0.031 (0.103)
501–1,000	0.071 (0.085)	−0.091 (0.129)	0.174 (0.095)	0.094 (0.102)	0.072 (0.119)	0.019 (0.131)
Hospital-level nurse staffing grade (ref: 1^st^ grade)						
2^nd^ to 5^th^ grades	−0.230 (0.107)^*^	−0.178 (0.165)	−0.243 (0.121)^*^	−0.255 (0.128)	−0.195 (0.150)	−0.288 (0.165)
Adj. R^2^	0.105	0.061	0.129	0.035	0.047	0.087
F test	2.02	1.59	2.36	1.33	1.46	1.86
*p*-value	0.0076	0.0564	0.0011	0.164	0.099	0.016
*N*	184	192	193	188	194	192

SE (standard errors); PhD (Doctor of Philosophy).

* *p* < 0.05.

** *p* < 0.01.

The effects of nurse turnover on team cohesion are presented in Table 5 (Models 21–24). In the total model, nurse turnover was unrelated to team cohesion. However, in the subscale models, moderate levels of turnover negatively affected task cohesion (*β* = −0.876, SE = 0.352) compared with the zero six-month turnover rate with an adjusted R-squared of 0.089. Likewise, low levels of turnover decreased social cohesion (*β* = −1.043, SE = 0.370) compared with the zero six-month turnover rate with an adjusted R-squared of 0.071.

**Table 5:** Nurse turnover contributing to team cohesion.

Variables	Total	Task cohesion	Social cohesion	Individual attraction to the group
	Model 21	Model 22	Model 23	Model 24
	β (SE)	β (SE)	β (SE)	β (SE)
Intercept	7.343 (1.007)^**^	8.204 (1.184)^**^	7.054 (1.335)^**^	6.062 (1.457)^**^
Nurse turnover				
Six-month turnover rate (ref: 0%)				
1–14%	−0.502 (0.281)	0.050 (0.330)	−1.043 (0.370)^**^	−0.447 (0.404)
15–23%	−0.417 (0.299)	−0.876 (0.352)^*^	−0.298 (0.398)	0.253 (0.434)
24–50%	−0.478 (0.306)	−0.623 (0.358)	−0.449 (0.406)	−0.263 (0.441)
Nurse- and work-related characteristics				
Sex (ref: Female)				
Male	−0.247 (0.561)	0.087 (0.659)	−1.090 (0.745)	0.773 (0.813)
Age, years in 2022	−0.037 (0.023)	−0.036 (0.027)	−0.031 (0.030)	−0.047 (0.033)
Highest nursing education (ref: Associate’s degree)				
Bachelor’s degree	−0.442 (0.237)	−0.740 (0.279)^**^	−0.340 (0.315)	−0.046 (0.343)
Master’s degree or PhD/DNP in nursing	−0.457 (0.502)	−0.853 (0.590)	−0.466 (0.666)	0.351 (0.728)
Marital status (ref: Married)				
Widowed, divorced, separated	0.521 (0.947)	0.507 (1.114)	0.398 (1.258)	0.786 (1.373)
Never married	0.192 (0.232)	0.039 (0.273)	0.056 (0.309)	0.769 (0.337)^*^
Subjective health status (ref: Poor)				
Very good	0.517 (0.376)	0.110 (0.442)	0.650 (0.500)	1.090 (0.545)^*^
Good	0.550 (0.273)^*^	0.457 (0.321)	0.511 (0.363)	0.822 (0.396)
Fair	0.160 (0.242)	0.190 (0.285)	−0.072 (0.321)	0.603 (0.350)
Work type (ref: Other)				
Three--shift rotation	0.146 (0.343)	0.262 (0.404)	0.178 (0.456)	−0.141 (0.497)
Work experience in current hospitals	0.040 (0.024)	0.021 (0.028)	0.027 (0.032)	0.100 (0.034)^**^
Workload	−0.041 (0.026)	−0.030 (0.030)	−0.060 (0.034)	−0.022 (0.037)
Type of unit (ref: Medical-surgical)				
Medical	0.443 (0.237)	0.387 (0.279)	0.611 (0.315)	0.212 (0.343)
Surgical	0.074 (0.238)	−0.304 (0.279)	0.405 (0.315)	0.185 (0.343)
Hospital size (ref: 201–300)				
301–400	0.378 (0.317)	0.190 (0.373)	0.844 (0.421)^*^	−0.171 (0.460)
401–500	−0.285 (0.271)	−0.167 (0.319)	−0.079 (0.357)	−0.869 (0.390)^*^
501–1,000	−0.273 (0.344)	−0.150 (0.402)	−0.139 (0.457)	−0.852 (0.495)
Hospital-level nurse staffing grade (ref: 1^st^ grade)				
2^nd^ to 5^th^ grades	−0.349 (0.434)	−0.501(0.502)	−0.218 (0.576)	−0.248 (0.629)
Adj. R^2^	0.062	0.089	0.071	0.040
F test	1.60	1.90	1.70	1.38
*p*-value	0.0552	0.0139	0.0354	0.1347
*N*	192	193	193	194

SE (standard errors); PhD (Doctor of Philosophy).

* *p* < 0.05.

** *p* < 0.01.

### 3.4 Mediating effects of workgroup processes

In this study, we predicted that nurse turnover affects nurse outcomes through workgroup processes including nurse–nurse collaboration and team cohesion. Such mediating effects can be tested using three equations on (1) the effects of nurse turnover on nurse outcomes (Tables 2, 3), (2) the effects of nurse turnover on workgroup processes (Tables 4, 5), and (3) the combined effects of nurse turnover and workgroup processes on nurse outcomes (Tables 2, 3). According to Baron and Kenny (36), all of these effects should be significant when testing mediating effects. The effect of nurse turnover on nurse outcomes should reduce when workgroup processes are included in the model. As aforementioned, higher nurse turnover decreases job satisfaction. Compared with the zero six-month turnover rate, moderate and low levels of nurse turnover decreased team cohesion in the subscale model (task cohesion and social cohesion). The effects of higher nurse turnover on job satisfaction decreased when team cohesion was added into the model. Thus, the mediating effects of team cohesion were partially supported.

## 4 Discussion

Nurse turnover is a critical issue in health care and may be dysfunctional. To address this issue, this study investigated the relationship among nurse turnover, workgroup processes, and nurse outcomes using McGrath’s (12) IPO framework. The most important finding of this study was that nurse turnover negatively affected the job satisfaction of nurses and several subscales of team cohesion (task cohesion and social cohesion). Further, team cohesion partially mediated the relationship between nurse turnover and job satisfaction.

O’Brien-Pallas et al. (9) found a negative relationship nurse turnover rate (the one-year in their study) and job satisfaction. Similarly, we found a negative relationship between the six-month turnover rate and job satisfaction. Compared with the zero six-month turnover rate, high turnover adversely affected job satisfaction. A previous study did not find a relationship between turnover and team cohesion (13). However, Price (37) stated that interpersonal interactions are difficult to form in nursing units with high turnover. Our study provided empirical evidence of the negative relationship between nurse turnover and team cohesion. Further, the mediating effect of team cohesion on the nurse turnover–job satisfaction relationship examined in this study provided empirical evidence of the underlying mechanism of the relationship between nurse turnover and nurse outcomes. Price (37) also explained that turnover reduces consensus and increases conflict among workgroup members, thus reducing satisfaction among stayers and leading to turnover (25). However, in this study, the effect of nurse turnover on intent to leave among stayers was not found. Future studies are required to investigate whether nurse turnover stimulates further turnover. This study also found no evidence of the impact of nurse turnover on nurse–nurse collaboration in either the total or the subscale models. Therefore, further investigations are required to determine this relationship.

This study provided evidence that increasing nurse–nurse collaboration can improve job satisfaction and reduce intent to leave, emphasizing the significance of nurse–nurse collaboration for improving nurse outcomes. Nurse–nurse collaboration has garnered attention in healthcare as an important managerial strategy for improving healthcare outcomes (38). Nurses must constantly collaborate to provide nursing care for 24 h in hospitals (30). Ylitörmänen et al. (19) found a model to explain the positive effects of nurse–nurse collaboration on job satisfaction, consistent with the findings of our study. Collaboration among healthcare team members contributes to higher levels of job satisfaction among nurses (39). Ma et al. (18) provided evidence that better nurse–physician collaboration and nurse–nurse collaboration are related to higher job satisfaction and lower nurses’ intent to leave. These findings are consistent with those of the current study. Thus, nurse–nurse collaboration can be considered to be a significant strategy for improving nurse outcomes, such increasing job satisfaction and reducing intent to leave.

Team cohesion was also significantly associated with job satisfaction and intent to leave. Lee et al. (21) studied team cohesion among hospital nurses and found that it is negatively correlated with turnover intention. Penconek et al. (22) reviewed job satisfaction of nurse managers and found that workgroup/co-worker cohesion is a key determinant of job satisfaction among nurse managers. Moreover, team cohesion is considered to be a multidimensional construct, not only including an interpersonal attraction but also task commitment (31, 40). Both aspects of team cohesion can affect job satisfaction and intent to leave. Hence, team cohesion must be fostered among nurses.

This study had several limitations. Although we contacted all general hospitals with 201–1,000 beds in Korea, only 35 (13%) hospitals participated in this study, and data from 28 (10%) hospitals were used for the analysis. The generalizability of the study findings is thus limited. Further, there was potential for self-selection bias among the nurses who responded. Nurses more concerned about nurse turnover are more likely to address nurse turnover issues. Further, the mean age of the study sample (32.6 years) are younger than that of a national sample because we included staff nurses excluding nurse managers (41) Thus, the study findings should be interpreted with caution.

Another limitation is that all the data were collected from a self-reported survey, which can be subject to errors owing to recall and socially desirability bias. In particular, we collected the turnover data 6 months before the data collection time. Thus, this finding could be subject to recall bias.

Finally, as we used a cross-sectional design, no causal relationship between nurse turnover and nurse outcomes could be found. Because this study focused on the relationships among nurse turnover, workgroup processes, and nurse outcomes, other variables that may affect nurse outcomes were not considered. For example, the relationship between the nurse and patient could be examined in future studies of nurses’ job satisfaction. The properties of the hospitals that were not included in this study can be examined. Future studies could also address these limitations using a longitudinal study and various measurement methods, other than self-reporting.

## 5 Conclusion

This study examined the impact of nurse turnover on workgroup processes and nurse outcomes, as well as, the mediating effects of workgroup processes on the relationship between nurse turnover and nurse outcomes. Nurse turnover decreases nurses’ job satisfaction and team cohesion, especially task cohesion and social cohesion. Among workgroup processes, team cohesion partially mediates the relationship between nurse turnover and job satisfaction. Given the limited evidence on the impact of nurse turnover, this study provides evidence to support the significant adverse impacts of nurse turnover on nurse outcomes and the underlying mechanism to explain this relationship. Future studies should examine this relationship using a longitudinal design.

## 6 Implication for nursing and health policy

This study’s findings support the development of managerial planning and policies to reduce nurse turnover. Nurse turnover is harmful to nurses’ job satisfaction and detrimental to workgroup processes, such as team cohesion. Healthcare organizations must create a positive work environment to reduce turnover. States and countries should try to develop and establish nursing and health policies to prevent turnover. Mandating a nurse-to-patient ratio policy or banning mandatory overtime among nurses could be considered. At the same time, retention strategies should be developed to make hospitals provide nursing care services with quality and safety.

## Data availability statement

The raw data supporting the conclusions of this article will be made available by the authors, without undue reservation.

## Ethics statement

The studies involving humans were approved by Ewha Womans University Institutional Review Board. The studies were conducted in accordance with the local legislation and institutional requirements. The ethics committee/institutional review board waived the requirement of written informed consent for participation from the participants or the participants’ legal guardians/next of kin because using an online survey, a written informed consent was waived.

## Author contributions

S-HB: Conceptualization, Formal Analysis, Funding acquisition, Investigation, Methodology, Project administration, Supervision, Writing – original draft, Writing – review & editing. SK: Data curation, Formal Analysis, Methodology, Writing – original draft, Writing – review & editing. HM: Data curation, Methodology, Writing – review & editing.

## References

[1] Brook J Aitken L Webb R Maclaren J. Salmon D characteristics of successful interventions to reduce turnover and increase retention of early career nurses: a systematic review. Int J Nurs Stud. (2019) 91:47–59. doi: 10.1016/j.ijnurstu.2018.11.003, PMID: 30669077

[2] World, Health, & Organization (2020). State of the world’s nursing. Available at: https://www.who.int/publications/i/item/9789240003279 [Accessed July 10, 2023].

[3] Djupedal ILR Pallesen S Harris A Waage S Biorvatn B Vedaa O. Changes in the work schedule of nurses related to the COVID-19 pandemic and its relationship with sleep and turnover intention. Int J Environ Res Public Health. (2022) 19:8682. doi: 10.3390/ijerph19148682, PMID: 35886534 PMC9318054

[4] Labrague LJ de Los Santos JAA. Fear of COVID-19, psychological distress, work satisfaction and turnover intention among frontline nurses. J Nurs Manag. (2021) 29:395–403. doi: 10.1111/jonm.13168, PMID: 32985046 PMC7537256

[5] NSI Nursing Solutions, Inc (2022). 2022 NSI national health care retention & RN staffing report. Available at: https://www.nsinursingsolutions.com/Documents/Library/NSI_National_Health_Care_Retention_Report.pdf. [Accessed 11 February 2023].

[6] Kerzman H Dijk DV Siman-Tov M Friedman S Goldberg S. Professional characteristics and work attitudes of hospital nurses who leave compared with those who stay. J Nurs Manag. (2020) 28:1364–71. doi: 10.1111/jonm.13090, PMID: 32654342

[7] Hospital Nurses Association (KR) (2018). A survey on hospital nursing staffingBusiness report for hospital nurses association. Seoul: Hospital Nurses Association (2019).

[8] Halter M Boiko O Pelone F Beighton C Harris R Gale J. The determinants and consequences of adult nursing staff turnover: a systematic review of systematic reviews. BMC Health Serv Res. (2017) 17:824. doi: 10.1186/s12913-017-2707-0, PMID: 29246221 PMC5732502

[9] O'Brien-Pallas L Murphy GT Shamian J Li X Hayes L. Impact and determinants of nurse turnover: a pan-Canadian study. J Nurs Manag. (2010) 18:1073–86. doi: 10.1111/j.1365-2834.2010.01167.x, PMID: 21073578

[10] Park SH Boyle DK Bergquist-Beringer S Staggs VS Dunton NE. Concurrent and lagged effects of registered nurse turnover and staffing on unit-acquired pressure ulcers. Health Serv Res. (2014) 49:1205–25. doi: 10.1111/1475-6773.12158, PMID: 24476194 PMC4239846

[11] Bae S-H. Noneconomic and economic impacts of nurse turnover in hospitals: a systematic review. Int Nurs Rev. (2022) 69:392–404. doi: 10.1111/inr.12769, PMID: 35654041 PMC9545246

[12] McGrath JE. Social psychology. Rinehart and Winston: A Brief Introduction. Holt (1964).

[13] Bae S-H Mark B Fried B. Impact of nursing unit turnover on patient outcomes in hospitals. J Nurs Scholarsh. (2010) 42:40–9. doi: 10.1111/j.1547-5069.2009.01319.x 20487185

[14] Kozlowski SWJ Bell BS. Work groups and teams in organizations In: Borman WC Ilgen DR Klimoski RJ Weiner IB (Editor) Handbook of psychology: Industrial and organizational psychology, vol. 12. 2nd ed Hoboken, New Jersey: John Wiley & Sons, Inc (2013). 412–69.

[15] Ylitörmänen T Kvist T Turunen H. Perceptions on nurse–nurse collaboration among registered nurses in Finland and Norway. Scand J Caring Sci. (2019) 33:731–40. doi: 10.1111/scs.12669, PMID: 30866079

[16] Schot E Tummers L Noordegraaf M. Working on working together. A systematic review on how healthcare professionals contribute to interprofessional collaboration. J Interprof Care. (2020) 34:332–42. doi: 10.1080/13561820.2019.1636007, PMID: 31329469

[17] Kowalski MO Basile C Bersick E Cole DA McClure DE Weaver SH. What do nurses need to practice effectively in the hospital environment? An integrative review with implications for nurse leaders. Worldviews Evid-Based Nurs. (2020) 17:60–70. doi: 10.1111/wvn.12401, PMID: 31621192

[18] Ma C Shang J Bott MJ. Linking unit collaboration and nursing leadership to nurse outcomes and quality of care. J Nurs Adm. (2015) 45:435–42. doi: 10.1097/nna.0000000000000229, PMID: 26301550

[19] Ylitörmänen T Turunen H Mikkonen S Kvist T. Good nurse-nurse collaboration implies high job satisfaction: a structural equation modelling approach. Nurs Open. (2019) 6:998–1005. doi: 10.1002/nop2.279, PMID: 31367424 PMC6650654

[20] Mullen B Copper C. The relation between group cohesiveness and performance: an integration. Psychol Bull. (1994) 115:210–27. doi: 10.1037/0033-2909.115.2.210

[21] Lee J Kong J Lee H. A study on the team sharing spirit model, team effectiveness, team cohesion, team reliability, and turnover intention among hospital nurses. J Korean Soc Integ Med. (2020) 8:121–31. doi: 10.15268/ksim.2020.8.3.121

[22] Penconek T Tate K Bernardes A Lee S Micaroni SPM Balsanelli AP. Determinants of nurse manager job satisfaction: a systematic review. Int J Nurs Stud. (2021) 118:103906. doi: 10.1016/j.ijnurstu.2021.103906, PMID: 33765624

[23] Cox CA. Nurse manager job satisfaction and retention: a home healthcare perspective. J Nurs Manag. (2019) 50:16–23. doi: 10.1097/01.NUMA.0000558512.58455.68, PMID: 31247629 PMC6716559

[24] Ulrich B Barden C Cassidy L Varn-Davis N. Frontline nurse manager and chief nurse executive skills: perceptions of direct care nurses. Nurse Lead. (2019) 17:109–12. doi: 10.1016/j.mnl.2018.12.014

[25] Staw BM. The consequences of turnover. J Occup Behav. (1980) 1:253–73.

[26] Faul F Erdfelder E Lang AG Buchner A. G^*^power 3: a flexible statistical power analysis program for the social, behavioral, and biomedical sciences. Behav Res Methods. (2007) 39:175–91. doi: 10.3758/bf03193146, PMID: 17695343

[27] Brewer CS Kovner CT Greene W Cheng Y. Predictors of RNs’ intent to work and work decisions 1 year later in a U.S. national sample. Int J Nurs Stud. (2009) 46:940–56. doi: 10.1016/j.ijnurstu.2008.02.003, PMID: 18377910

[28] Shin S Oh SJ Kim J Lee I. Bae, S-H impact of nurse staffing on intent to leave, job satisfaction, and occupational injuries in Korean hospitals: a cross-sectional study. Nurs Health Sci. (2020) 22:658–66. doi: 10.1111/nhs.12709, PMID: 32144854

[29] Dougherty MB Larson EL. The nurse-nurse collaboration scale. J Nurs Adm. (2010) 40:17–25. doi: 10.1097/NNA.0b013e3181c47cd6, PMID: 20010373

[30] Lee Y-J Hwang J-I. Relationships of nurse-nurse collaboration and nurse-physician collaboration with the occurrence of medical errors. J Korean Acad Nurs Adm. (2019) 25:73–82. doi: 10.11111/jkana.2019.25.2.73

[31] Carless SA De Paola C. The measurement of cohesion in work teams. Small Group Res. (2000) 31:71–88. doi: 10.1177/104649640003100104

[32] Kang JH You SE. The team assessment and diagnostic instrument (TADI): validation of the Korean version and development of a brief form for disaster response teams. Crisis. (2017) 13:91–106. doi: 10.14251/crisisonomy.2017.13.5.91

[33] June KJ Choi ES. Reliability and validity of the Korean version of the Copenhagen psyco-social questionnaire scale. Korean J Occup Health Nurs. (2013) 22:1–12. doi: 10.5807/kjohn.2013.22.1.1

[34] Lawler EE. Satisfaction and behavior. In: Hackman JR Lawler EE Porter LW (Editors). Perspectives on behavior in organizations. 2nd ed New York: McGraw-Hill (1983), 39–50.

[35] Park K-O Kim JK Kim SY Chang S. A model on turnover intention of chief nurse officers. J Korean Acad Nurs. (2012) 42:9–18. doi: 10.4040/jkan.2012.42.1.9, PMID: 22410597

[36] Baron RM Kenny DA. The moderator-mediator variable distinction in social psychological research: conceptual, strategic, and statistical considerations. J Pers Soc Psychol. (1986) 51:1173–82. doi: 10.1037//0022-3514.51.6.1173, PMID: 3806354

[37] Price JL. The study of turnover. Ames, Iowa: Iowa State University Press (1977).

[38] Al-Ajarmeh DO Rayan AH Eshah NF Al-Hamdan ZM. Nurse–nurse collaboration and performance among nurses in intensive care units. Nurs Crit Care. (2022) 27:747–55. doi: 10.1111/nicc.12745, PMID: 34962022

[39] Yasin YM Kerr MS Wong CA Bélanger CH. Factors affecting nurses’ job satisfaction in rural and urban acute care settings: a PRISMA systematic review. J Adv Nurs. (2020) 76:963–79. doi: 10.1111/jan.14293, PMID: 31840301

[40] Grossman R Nolan K Rosch Z Mazer D Salas E. The team cohesion-performance relationship: a meta-analysis exploring measurement approaches and the changing team landscape. Organ Psychol Rev. (2022) 12:181–238. doi: 10.1177/20413866211041157

[41] Korea Ministry of Health and Welfare (2020). Results of the national survey of healthcare workforce. Available at: http://www.mohw.go.kr/react/al/sal0301vw.jsp?PAR_MENU_ID=04&MENU_ID=0403&CONT_SEQ=372084&page=1#:~:text=(%EA%B0%84%ED%98%B8EC82%AC)%20ED%8F%89%EA%B7%A0%EC%97%B0%EB%A0%B9%EC%9D%80%2036.2,%EC%97%90%EC%84%9C%203.3%EC%84%B8%EA% B0%80%20%EC%A6%9D%EA%B0%80%ED%95%98%EC%98%80%EB%8B%A4.&text=%EC%9D%98%EC%82%AC20ED%8F%89%EA%B7%A0%EC%97%B0%EB%A0%B9%EC%9D%98%20%EAB2BD%EC%9A%B0,%EC%B0%A8%EC%9D%B4%EB%8A%94%205.2%EC%84%B8EC9D%B4%EB%8B%A4 [Assessed October 02, 2023].

